# A Decrease in the Hardness of Feces with Added Glucosylceramide Extracted from Koji *In Vitro*—A Working Hypothesis of Health Benefits of Dietary Glucosylceramide †

**DOI:** 10.3390/life14060739

**Published:** 2024-06-07

**Authors:** Huanghuang Dai, Johan Hariwitonang, Nao Fujiyama, Chihiro Moriguchi, Yuto Hirano, Fumio Ebara, Shigeki Inaba, Fumiyoshi Kondo, Hiroshi Kitagaki

**Affiliations:** 1 The United Graduate School of Agricultural Sciences, Kagoshima University, 1-21-24, Korimoto, Kagoshima 890-0065, Kagoshima, Japan; 21975002@edu.cc.saga-u.ac.jp (H.D.); sk1015@cc.saga-u.ac.jp (F.E.); inabas@cc.saga-u.ac.jp (S.I.); kondof@cc.saga-u.ac.jp (F.K.); 2 Graduate School of Advanced Health Sciences, Saga University, 1, Honjo-cho, Saga City 840-8502, Saga, Japan; johanhariw090201@gmail.com (J.H.); 23626025@edu.cc.saga-u.ac.jp (C.M.);; 3 Faculty of Agriculture, Saga University, 1, Honjo-Cho, Saga City 840-8502, Saga, Japan

**Keywords:** koji, glucosylceramide, skin barrier, food function, feces, hardness

## Abstract

Skin barrier function, prevent colon cancer, head and neck cancer, and decrease liver cholesterol. However, the mechanism of action has not yet been elucidated. In this study, we propose a new working hypothesis regarding the health benefits and functions of glucosylceramide: decreased fecal hardness. This hypothesis was verified using an *in vitro* hardness test. The hardness of feces supplemented with glucosylceramide was significantly lower than that of the control. Based on these results, a new working hypothesis of dietary glucosylceramide was conceived: glucosylceramide passes through the small intestine, interacts with intestinal bacteria, increases the tolerance of these bacteria toward secondary bile acids, and decreases the hardness of feces, and these factors synergistically result in *in vivo* effects. This hypothesis forms the basis for further studies on the health benefits and functions of dietary glucosylceramides.

## 1. Introduction

### 1.1. History of Sphingolipid Research

Sphingolipids are a group of organic lipid molecules composed of sphingosine bases and free fatty acid residues. Sphingolipids are components of eukaryotic cell membranes [1]. Its chemical structure was first elucidated by Thudicum in 1874 [2]. In the 1930s, sphingolipids gained attention because of their relevance in neural diseases [3]. In the 1960s, the loss of enzymatic activity in sphingolipid biosynthetic pathways was elucidated [4]. In the 1980s, its relevance to cancer and its signaling pathways were elucidated [5]. From the 1990s, genes involved in biosynthetic pathways were identified [6]. In 1991, a decrease in ceramide levels in the skin was proposed as the cause of atopic dermatitis [7]. Around 2000, the use of dietary sphingolipids for the prevention of colon cancer was reported [8,9]. In the 2010s, an increase in the skin barrier function due to dietary sphingolipids was reported [10]. However, the mechanisms linking dietary sphingolipids to physiological functions remain unclear.

### 1.2. Molecular Diversity of Sphingolipids

Sphingolipids consist of sphingoid bases, sphingosine-1-phosphate, ceramides, phosphodiester-bound complex sphingolipids (such as sphingomyelin, IPC, MIPC, GIPC, and ceramide-phosphoethanolamine), and glycosyl-bound complex sphingolipids (such as glucosylceramide, galactosylceramide, and glycosylsphingolipids). Glucosylceramide is a sphingolipid composed of a sugar moiety and a ceramide [11,12].

### 1.3. Fate of Dietary Sphingolipids

Because sphingolipids are present in several foods [13,14], the fate of dietary sphingolipids has been studied. Dietary sphingomyelin is digested by intestinal alkaline SMase (Alk-SMase) and neutral ceramidase (N-CDase), and is eventually hydrolyzed to ceramides, phosphorylcholine, sphingosine, and fatty acids in the small intestine [15,16]. Sphingosine can be absorbed by the intestinal mucosal cells and transformed into sphingosine 1-phosphate (S1P), ceramides, sphingomyelin, and glycosphingolipids [15,16,17,18]. Most of the ingested glucosylceramide is not degraded in the small intestine, and 50–90% of the glucosylceramide in the large intestine is excreted in feces [19]. Plant-derived glucosylceramides are hydrolyzed into free sphingoid bases in the digestive tract before being absorbed into the lymph. The recovery rate of sphingadienine from the small intestine is 0.18% [20]. 

### 1.4. Health Benefits and Functions of Glucosylceramides

Dietary glucosylceramide has been reported to prevent colon preneoplastic lesions [21], prevent head and neck cancers [22,23], inhibit colorectal cancer [24], inhibit throat cancer [25], inhibit inflammatory bowel disease (IBD) [26,27,28], improve atopic dermatitis, maintain skin moisture [29], improve lipid metabolism [26,30], improve cholesterol metabolism [31], improve intestinal microbial flora [32,33], and relieve bile acid pressure [34]. 

### 1.5. Mechanism of the Health Benefits and Functions of Glucosylceramide

The mechanisms underlying the health benefits and functions of glucosylceramides have been studied, and several mechanisms have been proposed. First, because the degraded products of sphingolipids exert metabolic effects on cultured cells [35,36,37], it has been proposed that trace amounts of absorbed degraded products of sphingolipids reach the skin and exert physiological effects [38]. One criticism is that the absorption efficiency is low. Further studies are required to test these hypotheses. Another study has described the *in vitro* anti-inflammatory effects of glucosylceramide *in vitro* [39]. This explains the prevention of colitis and colon cancer *in vivo*. Another study described the degradation of glucosylceramide in the colon by intestinal bacteria and the prevention of inflammation in the colon by the degraded ceramide [40]. Therefore, the presence and concentrations of intestinal bacteria in humans should be investigated. Although these mechanisms have been proposed, a direct mechanism remains unclear.

Therefore, to propose a new mechanism underlying the health benefits and functions of glucosylceramide, we performed an *in vitro* test on the hardness of feces supplemented with glucosylceramide. The hardness of feces decreased with the addition of glucosylceramide. This result led to a new working hypothesis regarding the mechanism of dietary glucosylceramide in decreasing the hardness of feces.

## 2. Materials and Methods

### 2.1. Materials

The feces used in the experiment were randomly obtained from cattle at the Saga University ranch. Koji was purchased from Tokushima Seiko (Tokyo, Japan). Soybean glucosylceramide was purchased from Nagara Science Co., Ltd. (Gifu, Japan). The Fall Cone Apparatus was purchased from Nishinihonshikenki Corporation (Osaka, Japan).

### 2.2. Extraction and Purification of Glucosylceramide

#### 2.2.1. Extraction of Lipids from Yellow Koji

Glucosylceramide was extracted and purified as previously described [41]. Koji (300 g) was homogenized using an agitator. Chloroform (200 mL) and methanol (400 mL) were added, and the mixture was dispersed by ultrasonication for another 30 min. Afterwards, chloroform (200 mL) was added, and the solution was sonicated for another 30 min. The solution was filtered through a PTFE mesh filter and qualitative filter paper (No. 2; Advantec Toyo Corporation, Tokyo, Japan) to obtain the extract. The extracts were then concentrated using a rotary evaporator (n-1110, Tokyo Science and Technology Co., Ltd., Tokyo, Japan). Then, a potassium hydroxide/methanol solution (0.8 M, 160 mL) was added, and put into a 42 °C thermostatic water tank (bw101, Yamato scientific Co., Ltd., Tokyo, Japan). The samples were shaken for 1 h. Afterward, chloroform (160 mL) was added first, followed by distilled water (144 mL), and the mixture was transferred to a funnel. It was allowed to stand at 4 °C for 12 h. The lower portion of the solution was recovered and concentrated using a rotary evaporator.

#### 2.2.2. Purification of Glucosylceramide from Koji by Open Column Chromatography

The lipids purified from koji were dissolved in 10 mL of a chloroform/methanol (2:1 *v*/*v*) solvent and introduced into a chromatographic tube (column length: 300 mm, inner diameter: 30 mm, with filter, Teflon Cock, As one Co., Ltd., Osaka, Japan) packed with silica gel for chromatography (silica gel 60, 70–230 mesh). Ethyl acetate/methanol (9:1 *v*/*v*) was used as the mobile phase and passed through the column in a total volume of 400 mL. The initial eluent (100 mL) was discarded as a waste liquid. The remaining 300 mL of the solution was collected. Ten milliliters of the solution was recovered from each test tube, and the 30 test tubes were labeled according to the order of solution outflow. Crude purified lipids were concentrated under reduced pressure using a tube evaporator (TVE-1100; Tokyo Physicochemical Instrument Co., Ltd., Tokyo, Japan). The concentrated lipids were dissolved in 1 mL of chloroform/methanol (2:1 *v*/*v*). Glucose ceramide from soybean (4 ug/mL) was spotted as a standard onto a 20 × 10 cm thin-layer chromatography (TLC) plate (TLC glass plate silica gel 60, Merck millipore Inc., Billerica, MA, USA). The TLC plate was developed using a solvent mixture of chloroform/methanol/acetic acid/water (94.3:16.5:10.8:3.3 *v*/*v*/*v*/*v*). The TLC plate was placed in a glass-developing tank and allowed to develop for 10 min before being removed. Sulfuric acid of orcinol (70%) was uniformly sprayed onto the TLC plate and heated in an oven at 100 °C for 40 min to develop a color. The lipid composition with fewer impurities was selected based on the lipid bands detected by TLC. The solution with fewer impurities was concentrated under reduced pressure using a rotary evaporator and subsequently dissolved in chloroform/methanol (2:1 *v*/*v*) for a further analysis.

#### 2.2.3. Isolation and Purification of Glucosylceramide from Koji by Thin-Layer Chromatography

All samples were spotted onto a 20 × 20 cm TLC plate in volumes of 40 µL. The leftmost spot is used as a control with 5 µL of glucosylceramide from soybean (4 ug/mL). The plate was then divided into two sections: In one section (control), sulfuric acid of orcinol (70%) was sprayed, followed by heating in an oven at 100 °C for 40 min. The position of glucosylceramide was confirmed by color development. The silica gel from the section containing the identified glucosylceramide was carefully scraped off using a blade and collected in a test tube for recycling. The purified silica gel was dissolved in 3 mL of chloroform/methanol (2:1, *v*/*v*) and subjected to ultrasonic treatment for 30 min. Centrifugation was performed using a Bench Centrifuge 5200 (Kubota Co., Ltd., Osaka, Japan) at 1500 rpm for 3 min. This process was repeated three times to ensure the removal of the residual silica gel from the solution.

#### 2.2.4. Detection of Glucosylceramide from Koji by Thin-Layer Chromatography

Glucosylceramide from soybean (4 μg/mL) was used as the standard substance and spotted to the TLC plate at varying volumes: 20, 30, and 40 µL. On the right side of the TLC plate, the glucosylceramide solution (4 μg/mL) was spotted at the following volumes: 4, 8, 12, 16, and 20 µL. Subsequently, the TLC plates were placed in a development tank containing a saturated TLC developing solvent (chloroform/methanol/acetic acid/water solution, 94.3:16.5:10.8:3.3 *v*/*v*/*v*/*v*) for a development period of 10 min. The plate was then sprayed with a solution containing 70% orcinol–sulfuric acid. It was then heated in an oven at 100 °C for 40 min to induce color development and aid in the analysis.

#### 2.2.5. Determination of Glucosylceramide from Koji by ImageJ

Using ImageJ (v1.52a), freely available software from NIH, USA, the bandwidth and range of the bands were calculated. For this analysis, soybean glucosylceramide was introduced onto the same TLC plate along with the lipid sample. The calibration curve was established, denoted by the equation y = a × √X, where ‘X’ represents the concentration of the band, ‘y’ symbolizes the standard quantity of glucosylceramide in the standard, and ‘a’ stands for a constant factor within the equation. This calibration curve was used to determine the quantity of glucosylceramide in the lipid samples based on the analysis of the observed color band.

### 2.3. Sample Processing

Initially, feces were autoclaved. The feces were then divided into two equal parts, each approximately 170 cm^3^ in volume. The previously purified glucosylceramide solution (0.85 mL, 4 µg/mL) was added to one group, and the concentration was adjusted to 20 µg/mL followed by thorough mixing.

### 2.4. Fall Cone Test 

The feces were molded into cylinders with a diameter of 6 cm and height of 2 cm (n = 3). The fall cone weighed 60 g, and its cone angle was 60° (Japan JGS 0142 [42]). The tip of the cone was brought close to the top surface of the feces, and the button was pressed to allow the cone to fall freely due to gravity. Three separate experiments were conducted on different parts of the same plane as that of the cylindrical sample.

### 2.5. Statistical Analysis

The statistical significance of differences was determined using Student’s *t* test (unpaired, one-sided).

## 3. Results

### 3.1. Measurement of Water Content of Feces

Because the water content affects the fluid behavior of feces, the water content of feces was measured. The water content of feces was approximately 83% in all feces and was relatively constant. Therefore, it can be inferred that feces exhibit non-Newtonian fluid behavior, the flow of feces also needs to adhere to the principles of non-Newtonian fluid behavior, and the relationship between shear stress and the shear strain rate in feces is not linear.

### 3.2. Extraction and Purification of Glucosylceramide from Koji

**Figure 1:**
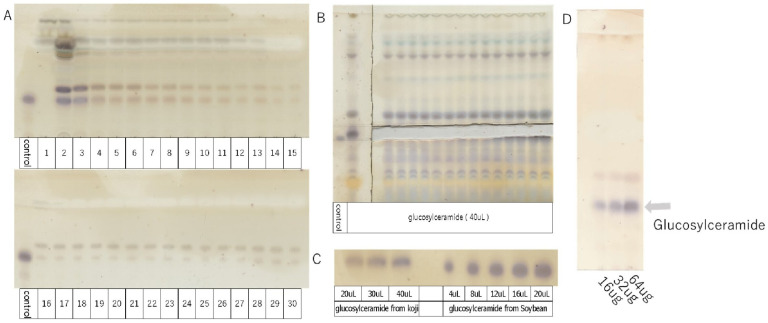
TLC analysis of glucosylceramide purified from koji (control = 5 µL).

The extraction and purification of glucosylceramide were performed using open column chromatography (Figure 1A) and thin-layer chromatography (TLC) (Figure 1B). Using glucosylceramide derived from soybean as a standard, the concentration of glucosylceramide obtained from koji was assessed using ImageJ software (Figure 1C). Subsequently, the purified glucosylceramide was dissolved in DMSO, resulting in a solution with a concentration of 4 µg/mL, set aside for further use (Figure 1D). The concentration of glucosylceramide used in this experiment was set as 20 µg/mL, calculated based on its concentration of foods [11].

- TLC developing solvent: chloroform/methanol/acetic acid/water solution = 94.3:16.5:10.8:3.3 *v*/*v*/*v*/*v*.
- A: Purification of glucosylceramide from koji by open column chromatography (1–30). Ethyl acetate/MeOH (9:1 *v*/*v*).
- B: Isolation and purification of glucosylceramide from koji by thin-layer chromatography.
- C: Detection of glucosylceramide from koji by thin-layer chromatography.
- D: Purified glucosylceramide solution configured with 4 µg/mL, showing 16, 32, and 64 µg schematics on TLC.

### 3.3. Decrease in the Hardness of Feces Added with Glucosylceramide

**Figure 2:**
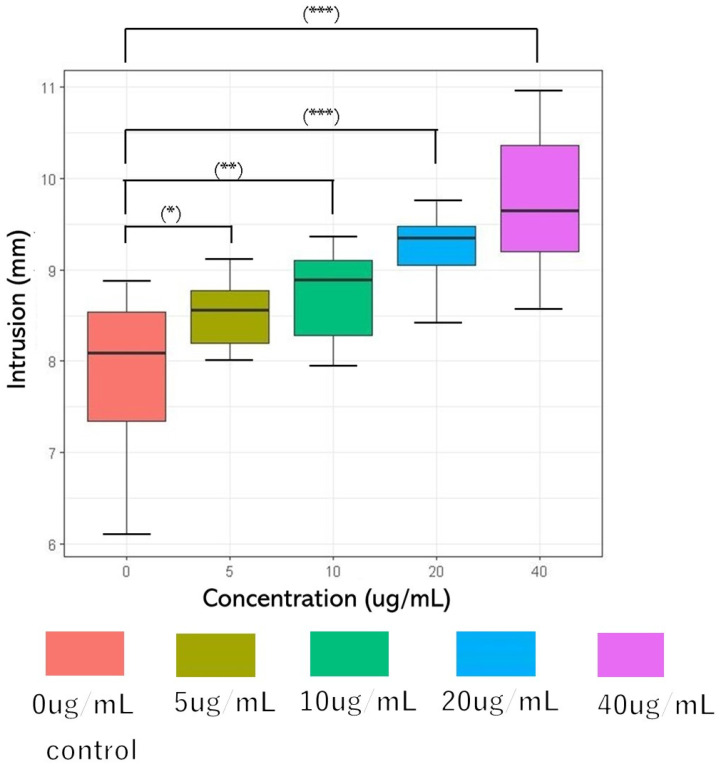
Cone penetration after adding glucosylceramide to feces. From left to right, the concentrations of glucosylceramide were 0 (red), 5 (ochre), 10 (green), 20 (blue), and 40 (purple) µg/mL, respectively. The penetration depths of each group were 7.865 mm (SD = 0.902), 8.515 mm (SD = 0.361), 8.727 mm (SD = 0.507), 9.215 mm (0.428), and 9.709 mm (SD = 0.808) (n = 10). In the related test, the fall cone weighed 60 g, and its cone angle was 60°. Pairwise comparison was performed using Tukey’s honest significant difference (Tukey’s HSD) test (* *p* < 0.05, ** *p* < 0.01, and *** *p* < 0.001).

The Bristol Stool Form Scale (BSFS), a 7-point scale, is widely used to measure the stool form [43]. However, it is a sensory evaluation of feces [44,45,46,47] and it is difficult to obtain objective data. Therefore, the fall cone test method of the hardness puncture experiment for food and soil mechanics experiments [48,49] was used in this study. The concentrations of glucosylceramide used in this experiment were 0, 5, 10, 20, and 40 μg/mL. The penetration depths were 7.865 mm (SD = 0.902), 8.515 mm (SD = 0.361), 8.727 mm (SD = 0.507), 9.215 mm (SD = 0.428), and 9.709 mm (SD = 0.808) (n = 10). Because DMSO was used as the solubilizer for glucosylceramide, the same volume of DMSO was added to the 0 μg/mL group in the experiment. Parametric variables were assessed using a paired one-way analysis of variance (ANOVA), followed by Tukey’s post hoc test. A two-sided *p*-value < 0.001 was considered statistically significant. As the concentration of glucosylceramide increased, the cone depth increased (Figure 2). Because feces are non-Newtonian fluids, it can be considered that after adding glucosylceramide, the fluidity of feces increases, the shear strength decreases, and the hardness of feces is reduced.

## 4. Discussion

Although the health benefits and functions of glucosylceramide have been reported for a long time, the underlying mechanisms have not yet been elucidated. In this study, we propose a new mechanism by which dietary glucosylceramide passes through the small intestine, acts on intestinal bacteria, and decreases fecal hardness. Further mechanisms of action of dietary glucosylceramides should be investigated.

Glucosylceramides are present in several foods [50], crops [51], and fermented Japanese foods containing koji [11]. Therefore, the health benefits of glucosylceramide may work in supplements as well as in Japanese fermented foods [52,53]. 

**Figure 3:**
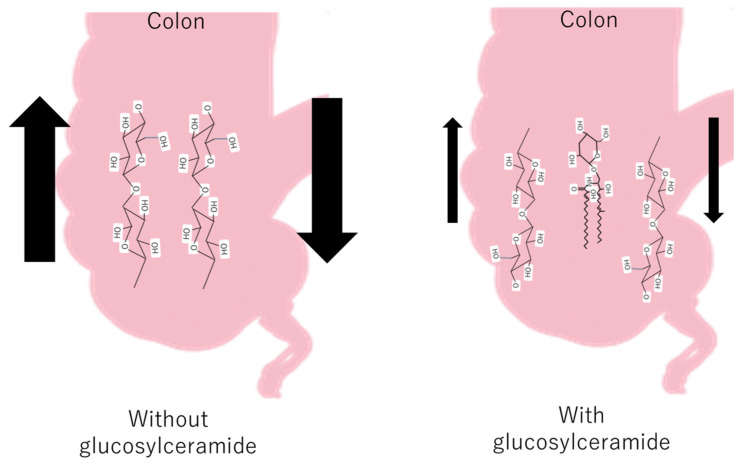
The hypothetical mechanism of the decrease in feces with and without glucosylceramide in the colon.

Some fecal hardness studies have shown a significant negative correlation between fecal hardness and fecal volume, fecal hardness, and fecal moisture [54], whereas fatty acid soaps are positively related to stool hardness [55]. The Bristol Stool Scale (BSFS) is widely used in clinical practice and research; however, it does not fully reflect the actual situation. As feces are mainly composed of residual cellulose, it can be inferred that glucosylceramide was inserted into the cellulose network and decreased the hardness of the colon (Figure 3). 

Several studies have shown that dietary glucosylceramide inhibits colon cancer and head and neck cancer [22], increases skin barrier function [10], decreases liver cholesterol [29], and alters intestinal microbiota [32]. It has been reported that a large portion of fed glucosylceramide passes through the small intestine without being degraded or absorbed [19,20]. However, several studies have shown that the degradation products of glucosylceramide, or glucosylceramide itself, upregulate metabolic genes or genes involved in skin barrier function [56]. Considering these facts, a small amount of absorbed glucosylceramide-degrading products may reach the liver or skin cells and exert these effects. However, this hypothesis requires further investigation. Other studies have shown that glucosylceramide is degraded by *Blautia glucerasei* in the large intestine and prevents colitis [40]. This may be the mechanism by which dietary glucosylceramide prevents colon cancer; however, the presence and amount of such bacteria await verification. The mechanism proposed in this study provides another independent explanation for the glucosylceramide paradox. 

Because a decrease in fecal hardness leads to the prevention of constipation, it is hypothesized that dietary glucosylceramide may exert its physiological effects by preventing constipation. Studies have shown that antipsychotic-induced constipation can interfere with sphingolipid metabolism, choline metabolism, and the sphingolipid signaling pathway [57]. However, these hypotheses should be verified in future studies.

Human and cow feces are physically different. Both human and cow feces contain solids consisting mainly of undigested and unabsorbed food residues, cellular debris, and other substances that cannot be absorbed by the body. Cattles are herbivores and their diet consists mainly of plants, whereas humans are omnivores and consume various diets that include both animal- and plant-based foods [58]. Consequently, there are significant differences between digestive systems. The digestive system of cattle is designed to digest cellulose from plants, which is more difficult to break down. The digestive system of cattle is longer than that of humans, which facilitates more efficient cellulose decomposition [59]. Cattle have four stomachs (rumen, reticulum, omasum, and abomasum) and rely on microbial fermentation in the stomach for cellulose breakdown. In contrast, humans have only one stomach and rely more on enzymes in the stomach and small intestine to aid food digestion [60]. These differences reflect the unique adaptations of each species to specific dietary and digestive requirements. Cow feces primarily consist of lignin, cellulose, and hemicellulose [61,62]. Therefore, cow feces can be used as a fuel source [62]. To the contrary, solid parts of human feces consist of proteins, fats, fibers, bacterial biomass, inorganic materials, and carbohydrates [63,64]. Their chemical and physical properties vary considerably depending on the health and diet of the individuals, with live and dead bacteria accounting for 25–54% of the dry weight of feces [63,64]. The median water content of feces is approximately 75% [63,64]. Normally, the diameter of the feces is comparable to that of the rectum [64], and as a result, their shapes are generally different. It is particularly interesting to note that feces from herbivores have a higher cellulose content and tend to float on the surface of water, whereas feces from carnivores do not float at all in water because of the high amount of indigestible residues in the feces [65]. Some studies have shown that the level of *Escherichia coli* in cow feces is higher than that in human feces [66]. Therefore, significant differences exist between cow and human feces regarding both the species and content of intestinal bacteria, as well as the composition, color, shape, and hardness of the feces. These differences not only reflect the physiological characteristics and digestive processes of different organisms, but also yield important information regarding biodiversity and ecological balance. 

The metabolic fate of lipids, especially triglycerides and cholesterol, in the intestine has been extensively studied. Following ingestion, triglycerides undergo emulsification with bile salts and hydrolysis by pancreatic lipases in the duodenum, yielding free fatty acids and monoglycerides. These products are absorbed by enterocytes and re-esterified into triglycerides and cholesterol esters. These lipids are subsequently assembled into chylomicrons, which are large lipoprotein particles that facilitate their transport to peripheral tissues via the lymphatic system for utilization or storage [67]. 

Dietary cholesterol is absorbed in the small intestine through a complex interplay between transporters and membrane proteins. One transporter, NPC1L1, is localized on the brush border membrane of enterocytes and facilitates cholesterol uptake from micelles in the intestine. Once absorbed, cholesterol undergoes esterification into cholesterol esters and is incorporated into chylomicrons for transport to the peripheral tissues or the liver. The regulation of cholesterol absorption involves factors such as dietary composition, bile acid metabolism, and genetic variations in NPC1L1 expression [68]. 

In contrast, sphingolipid degradation and absorption were low. Although sphingolipids undergo emulsification by bile salts, similar to other lipids, the efficiency of glucosylceramide degradation is low [69]. In addition, glucosylceramide from plant sources is excreted by P-glycoproteins even if it is absorbed by intestinal mucosal cells [19]. Therefore, the degradation and absorption of glucosylceramide in the small intestine are low, and most of the ingested glucosylceramide is transported to the large intestine. Therefore, the ingested glucosylceramide can be regarded as the concentration of glucosylceramide in the large intestine. The average ingestion of glucosylceramide is reported to be 26–77/mg (per day and capita, 60 kg weight human) [70]. The average amount of feces per day is approximately 128 g [64]. Therefore, the average concentration of glucosylceramide in the large intestine is estimated to be 200–600 μg/g. If we hypothesize that the gravity of feces is approximately 1 g/mL, this concentration is notably higher than the concentration of glucosylceramide used in this study (4 μg/mL), and the softening effect of glucosylceramide on feces can be regarded as sufficiently practical.

This study has several limitations. First, the concentration dependence of glucosylceramides has not yet been fully studied. This is because the amount of purified koji glucosylceramide was limited. To ensure the number of replicates for the three independent experiments, only several concentrations of glucosylceramide were used. This should be investigated further in future studies. Second, the results obtained with glucosylceramide were not compared with those of other lipids, such as sphingomyelin, ceramide, triglycerides, and cholesterol. However, this must be verified in future studies. Third, because of ethical concerns, this study used the feces of cows, not those of humans. However, the characteristics of the feces of cows and humans are considered similar. Fourth, the mechanism linking the decrease in fecal hardness to several physiological phenomena remains indirect. 

**Figure 4:**
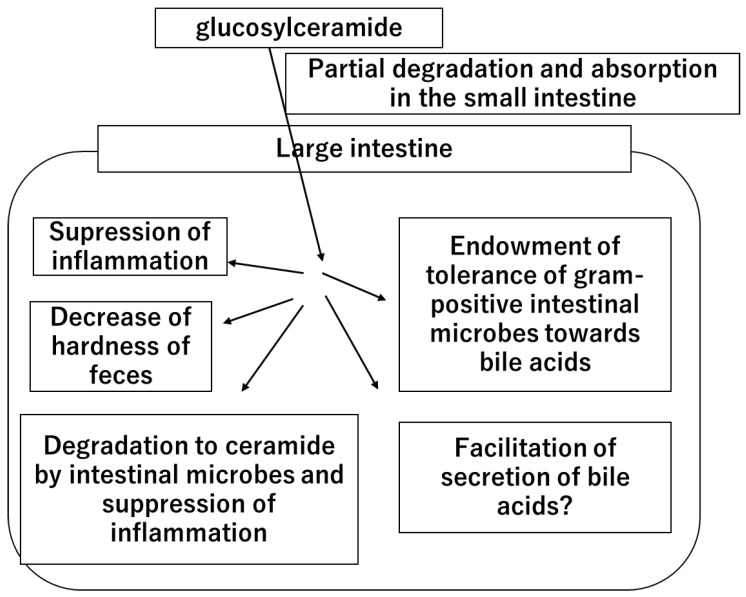
The hypothetical mechanism of the health functions and benefits of dietary glucosylceramide.

In summary, this study provides new insights into the role of glucosylceramide in the colon by decreasing fecal hardness. Given that glucosylceramide increases the tolerance of intestinal bacteria to secondary bile acids and promotes the production of lactic acid by intestinal bacteria [34], a new hypothesis regarding the role of glucosylceramide was considered (Figure 4). 

Once dietary glucosylceramide is ingested, a part of it is degraded and absorbed in the small intestine. A large portion of ingested glucosylceramide reaches the large intestine. In the large intestine, glucosylceramide acts on the intestinal cells and suppresses inflammation. Part of the glucosylceramide is degraded by intestinal microbes, generating ceramide and suppressing inflammation. Glucosylceramide acts on intestinal microbes, endows them with tolerance to bile acids, and facilitates lactic acid production. Glucosylceramide also facilitates bile acid secretion through an unknown mechanism. Glucosylceramide decreases the hardness of feces and prevents constipation.

## Data Availability

Inquiries can be directed to the corresponding author.
